# The Meal Type Rather than the Meal Sequence Affects the Meal Duration, Number of Chews, and Chewing Tempo

**DOI:** 10.3390/nu17091576

**Published:** 2025-05-03

**Authors:** Kanako Deguchi, Megumi Aoshima, Eri Hiraiwa, Chisato Ono, Chihiro Ushiroda, Risako Yamamoto-Wada, Mitsuyoshi Yoshida, Katsumi Iizuka

**Affiliations:** 1 Department of Clinical Nutrition, Fujita Health University, Toyoake 470-1192, Japan; kanasakuran@gmail.com (K.D.); 51021001@fujita-hu.ac.jp (M.A.); 51022099@fujita-hu.ac.jp (E.H.); chihiro.ushiroda@fujita-hu.ac.jp (C.U.); risako.wada@fujita-hu.ac.jp (R.Y.-W.); 2 Faculty of Medicine, Fujita Health University, Toyoake 470-1192, Japan; 3 Department of Medical Technology, Fujita Health University Haneda Clinic, Tokyo 144-0041, Japan; chisato.ono@fujita-hu.ac.jp; 4 Department of Dentistry and Oral-Maxillofacial Surgery, Fujita Health University, Toyoake 470-1192, Japan; mitsuyoshi.yoshida@fujita-hu.ac.jp; 5 Food and Nutrition Service Department, Fujita Health University Hospital, Toyoake 470-1192, Japan

**Keywords:** meal duration, chewing tempo, number of chews, number of bites, meal sequence, meal type, fast food, bento

## Abstract

Background/Aim: Obese individuals are often said to eat fast. Given that obese individuals are often reported to consume fast food more frequently, we hypothesized that fast food can be eaten more quickly. This study aimed to examine the effects of meal type on meal duration, considering meal sequence. Methods: Meal duration, number of chews and bites, and chewing tempo were measured among 41 participants (18 males, 23 females; average age, 41.1 years) using two meals: pizza (301 kcal) and hamburger steak bento (hamburger, rice and broccoli, 304 kcal; two eating sequences: vegetables first or last). Results: Compared with pizza, bento meals (vegetables first or last) were associated with longer meal durations (sec) (mean differences in pizza-bento (vegetable first): −182 [−245.6, −118.9], *p* < 0.0001; pizza-bento (vegetables last): −216.0 [−273.3, −158.7], *p* < 0.0001). In contrast, no differences in meal duration (sec) were observed regardless of the order of vegetable consumption (*p* = 0.14). These findings were consistent with the number of chews and chewing tempos across both sexes. The number of bites was similar among pizza, bento (vegetable fast), and bento (vegetable last). Meal duration was positively associated with the number of chews and bites and meal type and negatively associated with age and sex. BMI was not associated with meal duration. Conclusions: Meal type affected meal duration, the number of chews, and the chewing tempo, independent of meal sequence. To eat more slowly, it is important to pay attention to the meal type.

## 1. Introduction

Obesity is a condition that contributes to cardiovascular risk and malignant disease risk [1,2]. Various nutritional guidelines to prevent obesity have been proposed [3,4,5]. The basic principle of dietary therapy for obesity is to limit excess food intake while maintaining a balanced intake of nutrients. Common strategies for reducing energy intake involve avoiding carbohydrate- and fat-rich diets while increasing fiber consumption. However, individuals with obesity frequently consume ultra-processed foods, also called fast foods [6,7,8,9]. Fast foods, known for their mass production and affordability, are typically high in fat and sugar [10,11,12]. These components activate the brain’s reward system, inducing pleasure that can lead to dependency. Consequently, reducing the frequency of fast-food consumption is considered a preventive method against obesity.

Eating slowly is also believed to reduce food intake and prevent obesity [13,14,15,16]. Various methods to help people eat slowly have been devised [3,4,5]. However, the question of how to help people eat more slowly is actually a difficult one for both patients and the health care providers who explain it to them. Therefore, it is necessary to identify the factors that contribute to slow eating. To clarify the factors influencing meal duration, we previously reported that meal duration was positively associated with the number of chews and bites taken [17]. Furthermore, eating while listening to slow music with sound stimulation has been shown to lead to increases in meal duration, the number of chews, and the number of bites [17]. However, in our previous study, the test meal was only a pizza (ultra-processed foods and fast food), which was eaten without chopsticks. In Japan, we always use chopsticks except for fast food. Meals requiring chopsticks are likely to take longer to consume than those eaten with their hands. However, the empirical evidence supporting this assumption is insufficient. Therefore, we hypothesized that meal type may influence meal duration.

Meal sequences, especially Carbohydrate-last meal patterns, are often considered in the diets of patients with diabetes mellitus [18,19,20,21,22]. Carbohydrate-last meal sequences are known to reduce increases in blood glucose levels, among other factors [18,19,20,21,22]. Carbohydrate-last meal sequences are associated with increased mealtimes and reduced glycemic variability, which refers to swings in blood glucose levels when a continuous glucose monitoring system is used [22]. Incretin secretion is believed to be involved in this effect on the stomach [18]. The first consumption of vegetables also decreases postprandial glucose levels [23,24]. Moreover, the habit of eating vegetables first is correlated with increased functional capacity in older adults with diabetes [25]. Thus, the meal sequence is potentially useful in nutritional guidance for preventing and addressing diabetes and obesity; however, its effects on meal duration and chewing tempo remain unclear.

Sex is also important when investigating eating behaviors and food preferences [17,26,27]. Men and women have different dietary preferences and eating habits [17,27,28]. For example, men consume meat more frequently than women do, and women consume fish more frequently than men do [28]. In addition, the meal duration and number of chews differ by sex [17]; therefore, it is important to analyze these factors separately for men and women [17,27,28].

The effect of age on eating behavior is also unknown. It has been reported that meal duration increases rapidly after the age of 60 years [29]. According to self-reported statistics for Japan, the daily meal durations for individuals aged 25–34 years, 35–44 years, 45–54 years, 55–64 years, 65–74 years, and 75 years and older were 84, 88, 100, 107, 115, and 125 min, respectively [29]. Differences by age were characterized by particularly long breakfast times for elderly individuals (16, 17, 23, 26, 33, and 36 min for individuals aged 25–34 years, 35–44 years, 45–54 years, 55–64 years, 65–74 years, and 75 years and older, respectively) [29]. In contrast, according to the Ministry of Health, Labor and Welfare’s 2016 Dental Disease Survey in Japan, the number of teeth among individuals aged 25–34 years, 35–44 years, 45–54 years, 55–64 years, 65–74 years, and 75 years old and older decreased to 28.7, 28.2, 27.0, 24.5, 20.8, and 15.7 teeth, respectively [30]. In addition, it could be expected that the number of teeth decreases after middle age, so that people eat without chewing well; therefore, their meal duration is rather short, but the cause remains unknown. With respect to meal duration, no data are available to compare the meal durations of younger people and older people eating the same meal; therefore, the relationship between meal duration and age is unknown. Therefore, in addition to meal type, meal sequence, and sex, the effect of age on meal duration is also unresolved.

In this study, we clarify the effects of different meal types (pizza and hamburger steak bento) and meal sequences (vegetable first vs. vegetable last) on meal duration in male and female individuals aged 20–65 years. Experiments involving eating pizza and hamburger steak bento consisting of a hamburger, rice, and broccoli (vegetable first, vegetable last) were each spaced 4 weeks apart. Meal duration, number of chews, number of bites, and chewing tempo were measured for each meal. Our study suggests that appropriate selection of meal type is essential to nutritional guidance for obese individuals because meal type affects not only diet quality but also meal duration.

## 2. Materials and Methods

### 2.1. Participants

This study was a prospective intervention trial in which 41 participants aged 20–65 years (male: *n* = 18; female: *n* = 23) were recruited from the faculty and staff of Fujita Health University. The study was conducted from 10 July 2024 to 17 October 2024. To use this study’s results to guide our employees’ future health, we recruited individuals who actually work at the hospital. Consequently, we applied the following criteria. The study’s inclusion criteria were as follows: 1. Individuals (employees and students) who provided written consent to participate after being informed and demonstrating comprehension; 2. The participants were aged 20–65 years at the time of consent. Participants deemed by a physician to be unsuitable for participation in this study were excluded. Considering the very limited number of staff over 65 years of age and the impact of age, they were excluded from this recruitment. Although not included in the exclusion criteria, perhaps due to the small number of participants, those with anorexia nervosa, bulimia nervosa, BMI > 30, or preexisting diabetes or kidney disease were not included. The study was conducted according to the principles of the Declaration of Helsinki and was approved by the Research Ethics Committee of Fujita Health University (approval number HM23-472; approval date: 7 March 2024). All patients provided written informed consent before enrollment in the study. This study was registered with the UMIN Clinical Trial Registry (UMIN000054480; registered on 27 May 2024).

### 2.2. Measurement of Meal Duration, Numbers of Chews and Bites, and Chewing Tempo

The bitescan^TM^ device (Sharp Inc., Sakai, Osaka, Japan) was used to assess the meal duration, number of chews, number of bites, and number of chewing tempos in accordance with a previously established methodology [31,32]. In brief, meal duration was recorded by activating a stopwatch at the beginning and end of the meal, with verification conducted via video recording. The bitescan^TM^ apparatus (Sharp Inc., Sakai, Osaka, Japan) is designed to quantify the number of chews, number of bites, and number of chewing tempo [32,33]. Calibration was performed with bite-sized gummies. The skin behind the auricle moves as the jaw moves; the distance sensor in the bitescan^TM^ device is used to measure these small changes to determine the number of bites. The number of chews and the chewing tempo were determined via a proprietary algorithm [31,32].

### 2.3. Brief-Type Self-Administered Diet History Questionnaire (BDHQ)

Food frequency questionnaires are widely used to estimate the energy and nutrients in the diets of the population in clinical studies. The brief-type self-administered diet history questionnaire (BDHQ) is a self-administered diet history questionnaire used in Japan [17,33,34,35].

The BDHQ is written in Japanese, and questions are answered in the form of a mark sheet with numbers painted in with a pencil. The BDHQ can be completed within 20 min and consists of items related to the intake frequencies of 46 food and nonalcoholic beverage items; the daily intake of rice, including the type of rice (refined or unrefined) and miso soup; the frequency of alcoholic beverage consumption; the amount consumed for five types of alcoholic beverages; the usual cooking methods used by the respondent; and the general dietary behavior of the respondent [33,34]. On the basis of the answers to the food frequency questionnaires that the participants completed during the preceding month, we summarized the intake frequencies for approximately 100 nutrients and 58 food items. The estimated energy requirements (kcal) were calculated by multiplying the basal metabolic rate by the amount of physical activity performed by the respondent. Owing to the limitations of the questionnaire method, this value was approximately 20% lower than the value calculated on the basis of the dietary survey.

### 2.4. Handgrip Strength

Hand grip strength is an indicator of upper body strength, but is also used as an indicator of overall muscle strength in population studies. In this study, grip strength was calculated as the average strength of both hands. Grip strength was measured with a TOEI LIGHT grip strength tester ST3 T1781 (Toei Light Inc., Souka, Saitama, 340-0022, Japan).

### 2.5. Experiments

**Figure 1:**
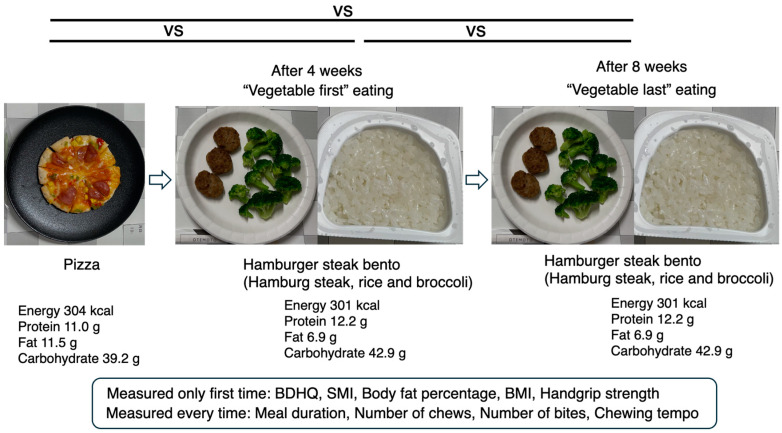
Outline of this experiment. On day 1, the body weight, BMI, body fat percentage, and skeletal muscle mass index (SMI) were calculated by dividing the skeletal muscle mass (kg) by the square of the height (m^2^), average grip strength of both hands, and BDHQ (Brief-Type Self-Administered Diet History Questionnaire) responses. The metrics for the first test meal (pizza, 304 kcal), including the number of chews, chewing tempo, and number of bites, were measured. The meal duration was measured via video analysis. Four weeks later, the metrics were measured again when the participants ate a hamburger lunch meal. The meal duration, number of chews, number of chewing tempos, and number of bites were measured when the participants were instructed to eat vegetables first. Four weeks later, a third set of measurements was taken as the participants again ate the hamburger lunch meal. The meal duration, number of chews, number of chewing tempos, and number of bites were measured when the participants were instructed to eat the vegetables last. A slice of pizza (Microwave Mix Pizza, Maruha Nichiro, Tokyo, Japan; energy, 304 kcal; protein, 11.0 g; fat, 11.5 g; carbohydrate, 39.2 g. Hamburger steak bento (hamburger steak (60 g), rice (100 g), and broccoli (100 g)) (energy, 301 kcal; protein, 12.2 g; fat, 6.9 g; carbohydrate, 42.9 g).

After ensuring that at least 4 h had passed since the participants had eaten breakfast, the BDHQ was administered, and the weight and handgrip strength of the participants were measured. Body weight, body fat percentage, and muscle mass were measured via InBody Dial H20N (Inbody Japan Inc., Tokyo, Japan). The skeletal muscle mass index (SMI) was calculated by dividing the limb skeletal muscle mass (kg) by the square of the height (m^2^). Body weight, body fat percentage, SMI, and grip strength were measured only at the first visit. Ten minutes later, the meal measurement experiment began (Figure 1). The participants first ate a slice of pizza (Microwave Mix Pizza, Maruha Nichiro, Tokyo, Japan; energy, 304 kcal; protein, 11.0 g; fat, 11.5 g; carbohydrate, 39.2 g). The pizza had a mass of 117 g and was 8 inches in diameter. Each pizza was precut into 12 equal portions for ease of eating. The meal duration, number of chews, average chewing tempo, and number of bites were measured. After 4 weeks, they were instructed to eat a hamburger steak bento (Japanese box lunch, “bento”) consisting of a hamburger steak (60 g), rice (100 g), and broccoli (100 g) (energy, 301 kcal; protein, 12.2 g; fat, 6.9 g; carbohydrate, 42.9 g). At this time, broccoli was eaten first, followed by rice and hamburger steak, and the meal duration, number of chews, average chewing tempo, and number of bites were measured. After 4 weeks, the bento was served in the order of rice, hamburger steak, and broccoli, and the meal duration, number of chews, chewing tempo, and number of bites were also measured (Figure 1).

### 2.6. Statistics

As this was an exploratory study, a predetermined sample size was not established. The values are presented as the means ± standard deviations (SDs). Comparisons between men and women regarding age, BMI, handgrip strength, the number of chews, and the food intake frequency survey results (total energy (kcal), protein (g), fat (g), carbohydrate (g), and dietary fiber (g) intake) were conducted via two-tailed t tests, and *p <* 0.05 was considered to indicate statistical significance.

Among the test meal groups (pizza, bento (vegetable first), and bento (vegetable last)), statistical analysis was performed via one-way ANOVA followed by Tukey post hoc tests. A *p*-value < 0.05 was considered to indicate statistical significance. The data are presented as the means (SDs).

A multivariate linear regression analysis was performed with meal duration as the dependent variable and the number of chews (times), chewing tempo (bpm), and number of bites as the independent variables adjusted for sex, age, and the type of test meal (pizza, bento (vegetable first), and bento (vegetable last)). Multivariate linear regression analysis was performed with meal duration as the dependent variable and the number of chews (Model 1), mean tempo (Model 2), and number of bites (Model 3) as the independent variables adjusted for sex, age, BMI, and meal type (pizza, 1; bento (vegetable first), 2; bento (vegetable last), 3). IBM SPSS software version 29.0.2 (IBM, Armonk, NY, USA) and GraphPad Prism version 10 (GraphPad Software Inc., San Diego, CA, USA) were used for statistical analysis.

## 3. Results

We previously reported that when a meal was pizza, meal duration, the number of chews, and the number of bites differed by sex. However, it is unclear whether the same results hold true for other diets. This study examined whether the type and order of meals can prolong meal duration.

First, we present the demographic and clinical data of the individuals who participated in this study. There was no difference between males (*n* = 18) and females (*n* = 23) in terms of age (40.3 ± 10.2 vs. 41.8 ± 10.3 years, *p* = 0.86). In addition, the average BMI values (kg/m^2^) were 23.9 ± 3.6 and 21.6 ± 2.1 for the male and female groups, respectively, with males having greater BMI values than females did (*p* = 0.026). The body fat percentage (%), skeletal muscle index (SMI) (kg/m^2^), and grip strength (kg) were also greater in men than in women (body fat percentage (%BW): 22.3 ± 7.0 vs. 31.2 ± 6.3, *p* < 0.001; SMI (kg/m^2^): 10.3 ± 1.0 vs. 7.9 ± 0.6, *p* < 0.001; and grip strength (kg): 41.1 ± 5.4 vs. 24.5 ± 4.0, *p* < 0.001) (Table 1).

The results of the BDHQ revealed that the estimated target energy levels for males and females were 2581.1 ± 98.1 kcal and 1974.6 ± 16.5 kcal (*p* < 0.001), respectively. The actual energy intake among the males and females was 1761.1 ± 619.6 kcal and 1545.9 ± 304.8 kcal, respectively, and did not differ between the sexes (*p* = 0.19). Furthermore, protein, fat, carbohydrate, and fiber intake did not differ between the sexes (*p* = 0.5, 0.9, 0.12, and 0.99, respectively). Thus, only BMI, body fat percentage, SMI, and grip strength differ according to sex.

**Figure 2:**
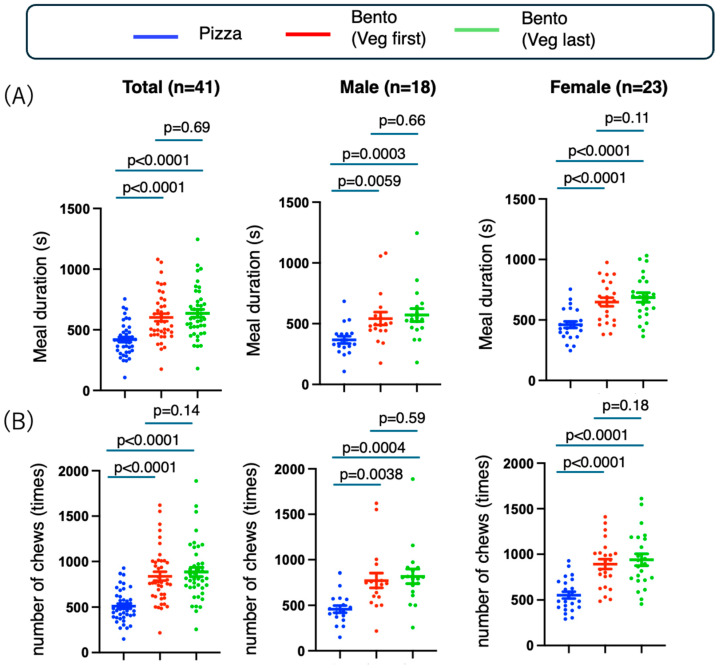

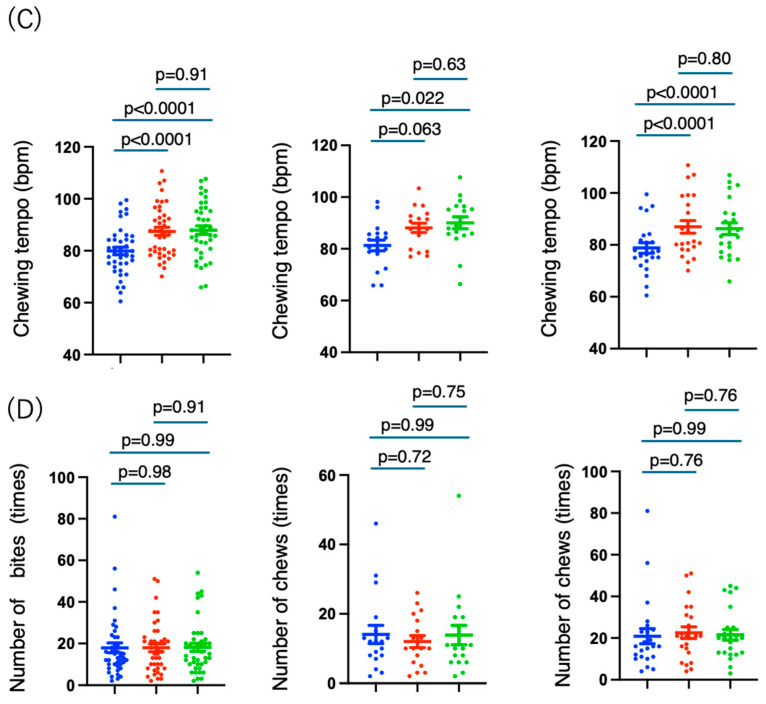
Effects of the test meal on the meal duration, number of chews, number of bites, and number of chewing tempos. (**A**) Meal duration, (**B**) number of chews, (**C**) chewing tempo, and (**D**) number of bites. The experimental procedure is shown in Figure 1. The chewing tempo, number of chews, and number of bites were measured via bitescanTM (Sharp Co. Ltd., Sakai, Osaka, Japan), and meal duration was recorded by activating a stopwatch at the beginning and end of the meal, with verification conducted via video recording. The meal types are represented as blue (pizza), red (Bento (vegetable fast), and green (Bento (vegetable last)).

We hypothesized that eating bento, which is served in pieces and eaten with chopsticks, would require longer mealtime than eating fast food, such as pizza, which is eaten with the hands. We compared three groups, pizza, bento (vegetable first), and bento (vegetable after), and examined whether the meal duration, number of chews, chewing tempo, and number of bites were affected by the meal type (pizza vs. bento) and meal sequence (vegetable first or last) (Figure 2). In terms of meal duration (sec), pizza was associated with shorter meal durations than bento meals were (mean difference (sec): pizza-bento (vegetable first): −182 [−245.6, −118.9], *p* < 0.0001; pizza-bento (vegetable last): −216.0 [−273.3, −158.7], *p* < 0.0001) (Figure 2A). In contrast, there was no difference in meal duration according to the meal sequence (mean difference (sec): bento (vegetable first)-bento (vegetable last): −33.76 [−76.00, 8.49], *p* = 0.14) (Figure 2A). These results were similar for men and women (males: mean difference (sec) in pizza-bento (vegetable first): −175.4 [−300.2, −50.57], *p* = 0.0059; mean difference (sec) in pizza-bento (vegetable last): −204.5 [308.3, −100.7], *p* = 0.0003; mean difference (sec) in bento (vegetable first)-bento (vegetable last): −29.1 [−113.2, 55.02], *p* = 0.66; females: pizza-bento (vegetable first): −338.5 [−431.3, −245.6], *p* < 0.0001; pizza-bento (vegetable last): −386.2 [−492.3, −280.1], *p* < 0.0001; bento (vegetable first)-bento (vegetable last): −47.74 [−112.5, 17.06], *p* = 0.18) (Figure 2A). Thus, meal type rather than meal sequence prolonged meal duration.

Since the number of chews is strongly considered, similar results to meal durations are expected. In fact, the number of chews (times) was lower for pizza than for bento meals (mean difference (times): pizza-bento (vegetable first): −328.4 [−429.0, −227.8], *p* < 0.0001; pizza-bento (vegetable last): −374.8 [−471.3, −278.2], *p* < 0.0001) (Figure 2B). There was no difference in the number of chews according to the meal sequence (mean difference (times): bento (vegetable first)-bento (vegetable last): −46.4 [−104.8, 12.06], *p* = 0.14) (Figure 2B). These results were similar for men and women (males: mean difference (times): pizza-bento (vegetable first): −315.5 [−528.3, −102.7], *p* = 0.0038; mean difference (times): pizza-bento (vegetable last): −360.2 [−549.8, −170.6], *p* = 0.0004; bento (vegetable first)-bento (vegetable last): −44.67 [−159.1, 69.78], *p* = 0.59; females: mean difference (times): pizza-bento (vegetable first): −338.5 [−431.3, −245.6], *p* < 0.0001; mean difference (times): pizza-bento (vegetable last): −386.2 [−492.3, −280.1], *p* < 0.0001; mean difference (times): bento (vegetable first)-bento (vegetable last): −47.74 [−112.5, 17.06], *p* = 0.18] (Figure 2B). Thus, the meal type rather than the meal sequence affects the number of chews.

The effect of meal type on mastication tempo (chewing tempo) is expected to be small because mastication movements are fundamentally regulated by the rhythm generator in the brainstem reticularis. Consistent with our expectations, pizza had less chewing tempo than bento meals did (mean difference (bpm): pizza-bento (vegetable first) (bpm): −7.55 [−10.9, −4.2], *p* < 0.0001; pizza-bento (vegetable last) (bpm): −8.00 [−11.4, −4.4], *p* < 0.0001) (Figure 2C). There was no difference in chewing tempo according to the meal sequence (bento (vegetable first)-bento (vegetable last) (bpm): −0.46 [−3.1, 2.2], *p* = 0.91) (Figure 2C). The results differed slightly between men and women, with men showing a significant difference in chewing tempo between the pizza and bento (vegetable last) meals (mean difference (bpm): −8.74 [−16.3, −1.2], *p* = 0.022) and females showing a significant difference in chewing tempo between the pizza and both bento meals (mean differences; vegetable first (bpm): −8.14 [−11.1, −5.2], *p* < 0.0001; vegetable last (bpm): −7.4 [−11.0, −3.9], *p* < 0.0001). The order of eating (meal sequence) was not significantly different between the two groups (males: bento (vegetable first)-bento (vegetable last): −1.9 [−7.2, 3.4], *p* = 0.63; females: bento (vegetable first)-bento (vegetable last): 0.70 [−2.0, 3.4], *p* = 0.80) (Figure 2C). Thus, meal type rather than meal sequence also affects chewing tempo.

Since a positive association between meal duration and the number of bites has been reported, one would expect the number of bites to be affected by the type of meal. However, the number of bites for the pizza meal was not significantly different from that for the bento meals (mean differences (times); pizza-bento (vegetable first): −0.02 [−4.3, 4.2], *p* = 0.98; pizza-bento (vegetable last): −0.3 [−5.2, 4.6], *p* = 0.99) (Figure 2D). There was no difference in the number of bites according to the meal sequence (mean differences (times); bento (vegetable first)-bento (vegetable last) (times): −0.3 [−3.9, 3.4], *p* = 0.98). These results were similar for men and women (males: mean differences (times); pizza-bento (vegetable first): 2.1 [−4.7, 8.8], *p* = 0.72; pizza-bento (vegetable last): 0.17 [−8.7, 9.1], *p* = 0.99; bento (vegetable first)-bento (vegetable last): −1.9 [−8.6, 4.8], *p* = 0.75; females: mean differences (times); pizza-bento (vegetable first): −1.7 [−7.6, 4.3], *p* = 0.76; pizza-bento (vegetable last): −0.7 [−6.9, 5.6], *p* = 0.96; bento (vegetable first)-bento (vegetable last): 1.0 [−3.3, 5.3], *p* = 0.83]) (Figure 2D). Thus, neither meal type nor meal sequence affected chewing tempo.

In a previous study, we reported that factors influencing diet were associated with sex, the number of chews, and the number of bites. In this study, we also examined the relationships between meal duration and meal type. Therefore, we conducted a multivariate analysis of the factors affecting meal duration. In Model 1, meal duration was related to the number of chews and sex (number of chews: 0.6 [0.5, 0.6], *p* < 0.001; sex: −42.6 [−73.8, −11.4], *p* = 0.008) (Table 2). In Model 2, meal duration was not related to chewing tempo but was related to meal type, sex, and age (meal type: 105.8 [65.1, 146.4], *p* < 0.001; sex: −132.1 [−202.2, −62.2], *p* < 0.001; age: −5.3 [−8.6, −2.0], *p* = 0.002) (Table 2). Finally, in Model 3, meal duration was associated with the number of bites, meal type, sex, and age (number of bites: 6.8 [4.6, 9.0], *p* < 0.001; meal type: 107.0 [73.4, 140.6], *p* < 0.001; sex: −67.7 [131.9, −3.5], *p* = 0.039; age: −4.6 [−7.6, −1.7], *p* = 0.002) (Table 2). In contrast, BMI was not associated with meal duration in models 1–3 (*p* = 0.85, *p* = 0.19, and *p* = 0.29, respectively).

Thus, meal duration was associated with the number of chews and bites, meal type, sex, and age.

## 4. Discussion

Eating slowly is important in nutritional guidance for obesity, but how to eat slowly remains unestablished. We hypothesized that meal type (individual serving, use of chopsticks) would influence meal duration. In this study, we examined whether meal type (pizza vs. hamburger steak bento) affects meal duration, the number of chews, the number of chewing tempos, and the number of bites. Independent of sex and meal sequence, eating pizza was associated with a shorter meal duration, a lower chewing frequency, and a lower chewing tempo than hamburger steak bento. Meal duration was associated with the number of chews, number of bites, age, sex, and meal type, but not the chewing tempo. These results indicate that regardless of the meal sequence, eating foods served individually leads to increases in meal duration and the number of chews. In addition to increasing the number of chews and bites, the appropriate selection of meal type (bento rather than fast foods) can prolong meal duration.

In this study, the meal duration and number of chews were greater for the hamburger steak bento than for the pizza meal. Consistent with our data, another group’s randomized control study revealed that, compared with non-ultra-processed foods, ultra-processed foods reduce the number of chews [36]. Pizza is an ultra-processed food, but hamburger steak bento contains unprocessed foods such as broccoli and rice. The degree of food processing might have a more substantial effect on meal duration and the number of chews. Moreover, hamburger bento is served in pieces and eaten with chopsticks. Pizza is eaten with the hands, but the hamburger steak bento is eaten with chopsticks. A previous study reported that eating rice with chopsticks is associated with a higher chewing rate and a lower eating rate [37]. Thus, the choice of a bento meal with various side dishes is important not only for obtaining different nutrients but also for eating more slowly. The degree of food processing, meal preparation (regardless of whether an individual is served), and eating methods (chopsticks vs. hand) may affect meal duration and the number of chews.

In this study, meal sequence had no effect on meal duration. The meal sequence is believed to play an important role in suppressing elevated blood glucose levels [18,19,20,21,22]; however, its effect on meal duration remains unknown. Eating vegetables first or carbohydrate last has a significant reducing effect on postprandial blood glucose and insulin, regardless of eating speed [21,22,23,24,38]. Considering the effects of meal sequence on blood glucose levels, eating vegetables first and consuming carbohydrates last may be the best choice because blood glucose levels are less likely to increase if vegetables are eaten first. Future research should examine differences between vegetable eaters and non-eaters in terms of their eating patterns, meal duration, and glycemic variability in the real world.

Interestingly, the chewing tempo, which is not easily influenced by various factors, was slightly greater for the hamburger steak bento (hamburger steak, rice, and broccoli) than for pizza. The basic pattern of rhythmic jaw movements produced during mastication is generated by a central pattern generator (CPG) located in the pons and medulla [39]. The output of CPG neurons is modified by inputs from higher-level brain regions and feedback from sensory receptors [39]. During mastication, the most important primary afferent feedback is provided by periodontal receptors and jaw-closing muscle spindle afferents, which provide information about food hardness and elasticity and the contraction properties of the jaw-closing muscles, respectively [39]. In addition, chewing tempo can be affected by sound stimulation, such as metronomes [17]. Slow-tempo music increases eating time, the number of chews, and total chewing duration [17,40]. We have previously shown that the chewing tempo is fine-tuned by external sound stimuli via metronomes to the same degree as in the present study. Thus, our results suggested that the chewing tempo was constant but fine-tuned by various external factors, such as external rhythmic stimuli and meal type.

In the present study, a multivariate analysis with meal duration as the dependent variable confirmed our previous data that meal duration is associated with the number of chews, number of bites, and sex [17]. These findings suggest that increasing the number of chews and bites may prolong meal duration regardless of meal type. When consuming any type of food, it is crucial to take smaller bites, chew thoroughly, and ensure that the mouth is empty before the next portion is introduced. This practice helps prolong meal duration and prevents overeating.

In contrast, BMI was not associated with meal duration, regardless of meal type. Our former paper using a pizza as a test meal also reported that BMI was not associated with meal duration when a pizza was eaten [17]. Individuals with obesity often consume more fast food [6,7,8]. This is attributed to the high carbohydrate and fat contents in fast food, which are believed to be effective in activating the reward system [10,11]. Studies indicate that self-reported obese individuals eat more quickly [15,16], although this is not observed when diets are controlled for equivalence. Thus, obese individuals prefer consuming ultra-processed meals rather than various unprocessed foods, potentially leading to shorter meal durations. Future research should verify meal durations and contents in the everyday setting of obese individuals. Modifying meal choices may serve as an effective strategy for addressing obesity.

Finally, there was a negative association between age and meal duration. The results revealed that the older the participants were, the shorter the meal duration was. In contrast, a previous study reported that time spent eating increases with age: individuals aged 55–64 years and individuals 65 years or older spend more time eating than individuals aged 18–54 years do [20]. This difference could be because meal duration was determined on the basis of self-reported data rather than measured data. Another possibility is that the number of times a person chews a meal decreases with age, which is influenced by the decrease in the number of teeth retained with increasing age [21]. The greater the number of teeth is, the higher the rate of chewing. According to the 2009 National Health Survey in Japan, the rate of having 20 or more teeth is 100% for individuals aged 15–29 years, but the rate decreases to 80% for individuals aged 50–59 years [21]. Therefore, if we eat the same food, deterioration of the oral environment, such as the presence of dental caries, may lead to a shorter meal duration via a decrease in the frequency of chewing.

One limitation of this study is that it was an exploratory study, and the number of participants was not set; however, the number of participants was larger than that in previous studies [11] and was sufficient to discriminate differences between men and women. This is not a randomized, comparative study. However, the present study compared pizza and bento in the same group of participants, which is considered a better design than a randomized study comparing pizza-only eaters and boxed lunch-only eaters. The sufficient time interval of 4 weeks suggests that there would be little difference between the pizza and boxed lunch in that order and the boxed lunch and pizza in that order. Moreover, the pizza and hamburger steak bento differed in terms of whether chopsticks were used. We believe that one of the important points of this study is the difference in eating methods, such as eating by hand or using chopsticks. Serving food individually and eating with chopsticks may lead to longer mealtimes, which may also lead to differences in nutritional guidance methods in various countries. Moreover, this study did not assess the participants’ number of teeth or their oral condition. Notably, elderly individuals often experience a reduction in the number of teeth, which can lead to chewing difficulties. In older adults, there is a positive correlation between the number of remaining teeth and the frequency of chewing [41,42]. While it is generally assumed that individuals with fewer teeth tend to eat more slowly, studies such as the present one, which included participants up to 65 years of age, highlight the need for future research to explore meal duration and chewing frequency in older individuals and those with fewer teeth. The present study also did not consider the influence of psychological factors on eating time. Macht and Simons, et al. referred to the short amount of time spent on eating behaviors as a characteristic of emotional eating [43]. Although those with a history of apparent overeating or eating disorders were not included in the present study, an objective assessment such as the Dutch Eating Behavior Questionnaire may be necessary, especially when targeting obesity [44]. Since participants do not usually eat with digital devices, it is necessary to ascertain whether there is a relationship between eating time with a machine and actual eating time at a meal. Finally, how eating slowly affects health maintenance needs to be clarified in the future through prospective studies.

## 5. Conclusions

In the present study, compared with those of pizza, the eating time and number of chews were greater when hamburger meals were eaten. However, eating vegetables first or last had no effect on meal duration. Serving foods individually and eating with chopsticks may be effective in providing nutritional guidance for individuals with obesity because this strategy increases meal duration and the number of chews. The guidance to select bento over fast food is important because of its superior nutritional value, meal duration, and number of chews. Future research should also verify meal durations and contents in the everyday setting of obese individuals. To continue eating slowly to prevent obesity, we must not only take smaller bites and chew well when eating, but also pay attention to the food we choose. In addition, psychological aspects and environmental improvements, which were not examined in this study, should also be considered. Simply playing relaxing music in the staff cafeteria may have the effect of extending mealtime by relaxing the mind, in addition to increasing the chewing tempo and frequency of chewing. Finally, clarification of the factors that influence meal duration is important not only for obesity but also for malnutrition. For those with poor appetite, fast foods with varied nutritional values may help them obtain nutrients before they feel full. In fact, high-energy semi-digestible formulas supplemented with vitamins and other nutrients are already being used in medical care for people with low nutritional status. As more factors that influence mealtimes are discovered, it will be possible to provide more personalized dietary guidance for individuals with obesity and malnutrition.

## Figures and Tables

**Table 1:** Background of participants.

	Total (*n* = 41)	Male (*n* = 18)	Female (*n* = 23)	*p*
Age (years)	41.1 (10.1)	40.3 (10.2)	41.8 (10.3)	0.66
BMI (kg/m^2^)	22.6 (3.0)	23.9 (3.6)	21.6 (2.1)	0.026
Body fat percentage (%)	27.2 (7.9)	22.3 (7.0)	31.2 (6.3)	<0.001
SMI (kg/m^2^)	8.9 (1.4)	10.3 (1.0)	7.9 (0.6)	<0.001
Average handgrip strength (kg)	31.8 (9.5)	41.1 (5.4)	24.5 (4.0)	<.0001
Estimated Energy Requirements (kcal)	2240.9 (311.6)	2581.1 (98.1)	1974.6 (16.5)	<0.001
Total energy (kcal)	1640.4 (475.4)	1761.1 (619.6)	1545.9 (304.8)	0.19
Water (mL)	1595.8 (426.4)	1757.7 (386.2)	1469.1 (420.5)	0.028
Protein (g)	60.3 (17.8)	62.5 (21.9)	58.5 (13.9)	0.5
Fat (g)	52.2 (15.1)	52.5 (18.8)	51.9 (11.9)	0.9
Carbohydrate (g)	213.7 (74.6)	236.5 (97.4)	195.8 (44.9)	0.12
Dietary fiber (g)	10.0 (3.7)	10.0 (3.6)	10.0 (3.9)	0.99

The data are presented as the mean (SD). *p* < 0.05 between males and females was considered significant.

**Table 2:** Multivariate regression analysis of meal duration and other factors.

	Model 1		Model 2		Model 3	
	β (95%CI)	*p*	β (95%CI)	*p*	β (95%CI)	*p*
Number of chews (times)	0.6 [0.5, 0.6]	<0.001				
Chewing tempo (bpm)			0.6 [−2.6, 3.8]	0.73		
Number of bites (times)					6.8 [4.6, 9.0]	<0.001
Meal types Pizza:1; Bento (vegetables first):2; Bento (vegetables last):3	−1.4 [−20.6, 17.9]	0.89	105.8 [65.1, 146.4]	<0.001	107 [73.4, 140.6]	<0.001
Sex (Male:1)	−42.6 [−73.8, −11.4]	0.008	−132.1 [−202.2, −62.2]	<0.001	−67.7 [131.9, −3.5]	0.039
Age (years)	−1.1 [−2.6, 0.4]	0.16	−5.3 [−8.6, −2.0]	0.002	−4.6 [−7.6, −1.7]	0.002
BMI (kg/m^2^)	0.5 [−4.7, 5.8]	0.85	8.0 [−4.1, 20.1]	0.19	5.6 [−4.9, 16.1]	0.29

Multivariate linear regression analysis was performed with meal duration as the dependent variable and the number of chews (Model 1), chewing tempo (Model 2), and number of bites (Model 3) as the independent variables adjusted for meal type (Pizza: 1; Bento (vegetables first): 2; Bento (vegetables last): 3, sex (male:), age, and BMI.

## Data Availability

Some or all datasets generated during and/or analyzed during the current study are not publicly available but are available from the corresponding author upon reasonable request.

## Abbreviations

The following abbreviations are used in this manuscript:

BDHQ	Brief-Type Self-Administered Diet History Questionnaire
